# Zingerone alleviates acute seizures by reducing intrinsic hippocampal neuronal excitability in a rat model of temporal lobe epilepsy

**DOI:** 10.3389/fnins.2026.1809792

**Published:** 2026-04-10

**Authors:** Hong Ni, Wenkang Yang, Shu Han, Yuanyuan Wang, Haihu Ding, Tianren Song, Mingrui Zhang, Jiandong Yu, Rongjing Ge

**Affiliations:** 1 Laboratory of Brain and Psychiatric Disease, Bengbu Medical University, Bengbu, Anhui, China; 2 Functional Experiment Center, School of Basic Medical Sciences, Bengbu Medical University, Bengbu, Anhui, China; 3 Department of Pathology, Handan Central Hospital, Handan, Hebei, China; 4 Department of Neurosurgery, The First Affiliated Hospital of Bengbu Medical University, Bengbu, Anhui, China; 5 Department of Pathophysiology, Bengbu Medical University, Bengbu, Anhui, China; 6 Anhui Engineering Research Center for Neural Regeneration Technology and Medical New Materials, Bengbu Medical University, Bengbu, China

**Keywords:** anticonvulsant effect, hippocampus, intrinsic neuronal excitability, neuroinflammation, pilocarpine, temporal lobe epilepsy, zingerone

## Abstract

**Background:**

Epilepsy is a complex neurological disorder characterized by recurrent seizures. Neuroinflammation and excessive neuronal excitation are key pathogenic factors, but current therapies fail to target these mechanisms effectively, highlighting the need for novel therapeutic agents. Zingerone, a bioactive compound derived from ginger (*Zingiber officinale Roscoe*), exhibits anti-inflammatory, antioxidant, and neuroprotective properties. However, its acute anticonvulsant efficacy and underlying mechanism in temporal lobe epilepsy (TLE) remain unclear. This study aimed to investigate whether zingerone exerts anticonvulsant effects by modulating neuronal excitability.

**Methods:**

A lithium chloride-pilocarpine-induced acute TLE rat model was established. Rats were randomly assigned to control, pilocarpine, and zingerone treatment groups (75, 150, and 300 mg/kg, i.p.). Seizure activity was evaluated via behavioral scoring (Racine scale) and electroencephalography (EEG). Immunohistochemistry (IHC), hematoxylin-eosin (HE) staining, and immunofluorescence were used to assess hippocampal microglial/astrocytic activation and neuronal damage. Whole-cell patch-clamp recordings were performed to analyze intrinsic neuronal excitability and synaptic transmission in hippocampal CA1 pyramidal neurons.

**Results:**

Acute administration of zingerone (150 and 300 mg/kg) significantly reduced the number and duration of Racine stage IV/V generalized seizures. Zingerone dose-dependently inhibited microglial (IBA1^+^) and astrocytic (GFAP^+^) activation (*p* < 0.01 for 150 mg/kg; *p* < 0.001 for 300 mg/kg) and preserved neuronal integrity in the hippocampal CA1 region, as evidenced by reduced neuronal shrinkage, pyknosis, and increased NeuN^+^ neuron density. Electrophysiological recordings revealed that zingerone (10 μM) decreased the firing frequency of CA1 pyramidal neurons (*p* < 0.05), prolonged the inter-spike interval (*p* = 0.0026), reduced the action potential peak (*p* = 0.041), increased the rheobase current (*p* = 0.042), and increased afterhyperpolarization amplitude (*p* = 0.0001). Furthermore, Zingerone also modulated excitatory synaptic transmission onto CA1 neurons.

**Conclusion:**

Zingerone exerts acute anticonvulsant and neuroprotective effects in a TLE rat model by suppressing hippocampal neuroinflammation and reducing the intrinsic excitability of pyramidal neurons. These findings highlight zingerone as a promising natural compound for developing novel adjuvant therapies for drug-resistant TLE.

## 1 Introduction

Epilepsy is a global neurological disorder affecting approximately 50 million people worldwide, with temporal lobe epilepsy (TLE) accounting for 30–40% of adult cases (World Health Organization, 2024; Fisher et al., 2017). TLE is characterized by recurrent seizures originating from the temporal lobe, frequently associated with hippocampal sclerosis, neuronal loss, and synaptic reorganization (Patel et al., 2019). Despite the availability of multiple antiepileptic drugs (AEDs) such as carbamazepine and levetiracetam are available, approximately 30% of TLE patients exhibit drug-resistant, and existing therapies merely suppress seizure activity without modifying the underlying epileptogenic process (Ryvlin et al., 2026; Kwan et al., 2010). This therapeutic gap underscores the urgent need to identify novel compounds targeting the core pathophysiological mechanisms of TLE.

Neuroinflammation and neuronal excitability dysregulation are central to TLE pathogenesis and two interrelated driving factors in the pathogenesis of temporal lobe epilepsy (TLE) (Vezzani et al., 2016; Rana and Musto, 2018; Borowicz-Reutt and Czuczwar, 2020). Seizure-induced activation of microglia and astrocytes triggers the release of pro-inflammatory cytokines, such as interleukin-1β (IL-1β) and tumor necrosis factor-α (TNF-α), which disrupt the balance between excitatory (glutamatergic) and inhibitory (γ-aminobutyric acidergic) synaptic transmission (Zhang et al., 2022; Ramos-Moreno et al., 2024). Simultaneously, excessive neuronal excitation caused by ion channel dysfunction, synaptic plasticity changes, and neuronal network remodeling promotes synchronous firing, a hallmark of seizures (Kudryashov et al., 2007; Yang et al., 2018). Hippocampus is a region highly vulnerable to seizure damage in the brain, which plays a central role in TLE, with significant excessive excitation and loss of pyramidal neurons in the CA1 region (Patel et al., 2019; Swindell et al., 2020). Dual treatment targeting neuroinflammation and neuronal excitability represents a promising strategy for developing effective therapies for TLE.

Natural products have emerged as valuable sources of multi-targeted compounds for neurological disorders (Ahmad et al., 2022; Yang et al., 2020; C Sekhar et al., 2024; Sekhar et al., 2025; Kunjumon et al., 2022). Ginger (Zingiber officinale Roscoe) is a widely used medicinal and edible plant, and its bioactive components (gingerols, shogaols, zingerone) exhibit diverse pharmacological effects (Mao et al., 2019; Semwal et al., 2015). Zingerone (4-(4-hydroxy-3-methoxyphenyl)-2-butanone) is a major ginger-derived phenol with well-documented pharmacological properties, known for its anti-inflammatory, antioxidant, anti-apoptotic, and neuroprotective effects (Molavinia et al., 2024; Eriten et al., 2024; Tuncer et al., 2024; Olasehinde and Olaokun, 2025; Xu et al., 2025; Oviosun et al., 2025; Ram et al., 2021). Recent study demonstrated that, following oral administration in rats, zingerone is absorbed and systemically distributed. It rapidly crosses the blood–brain barrier (BBB) and is easily metabolized in both rats and humans, achieving decent concentrations in the blood (Li et al., 2019; Songvut et al., 2024). Furthermore, zingerone modulates neuronal electrophysiology by inhibiting voltage-gated sodium and calcium channels (Kudryashov et al., 2007; Wang et al., 2025; Lai et al., 2022) and suppresses neuroinflammation in models of neurodegeneration (Ramos-Moreno et al., 2024; Oviosun et al., 2025). However, its acute anticonvulsant potential in TLE and the specific effects on hippocampal neuronal excitability modulation remain unelucidated. Most previous studies have focused on the chronic administration of ginger constituents, leaving their efficacy unknown during acute epileptic seizures, which are a critical clinical condition (Rashid et al., 2021; Eriten et al., 2024).

Furthermore, although ginger-derived compounds like [6]-gingerol and [6]-shogaol have been shown to exhibit anticonvulsant effects (Hitomi et al., 2017; Kim et al., 2010, 2022b), zingerone’s unique chemical structure (a less lipophilic derivative of gingerol) suggests distinct pharmacological profiles, including improved bioavailability and reduced toxicity (Li et al., 2019; Songvut et al., 2024). Unlike existing AEDs that primarily target single ion channels, zingerone’s multi-targeted properties make it a promising candidate for addressing the complex pathophysiology of TLE.

In this study, we hypothesized that zingerone exerts acute anticonvulsant effects in TLE by attenuating neuroinflammation and reducing the intrinsic excitability of hippocampal neurons. With using a lithium-pilocarpine-induced TLE rat model (Rashid et al., 2021), we combined behavioral, electrophysiological, and histological approaches to: (1) evaluate the acute anticonvulsant efficacy of zingerone; (2) assess its effects on hippocampal neuroinflammation and neuronal survival; (3) clarify its impact on intrinsic neuronal excitability and synaptic transmission. Our findings provide novel insights into zingerone’s therapeutic potential and underlying mechanisms, paving the way for its development as a natural adjuvant therapy for drug-resistant TLE.

## 2 Materials and methods

### 2.1 Animals

Fifty male Sprague-Dawley (SD) rats (8 weeks old, 220–250 g) were purchased from Shandong Jinan Pengyue Experimental Animal Breeding Co., Ltd. (License No.: SCXK 2022-0006). Rats were housed under standard conditions (12-h light/dark cycle, 20–26°C, 50 ± 20% humidity) with free access to food and water, and acclimatized for 1 week before experiments. All procedures were approved by the Laboratory Animal Ethics Committee of Bengbu Medical University [Approval No. (2024) 474] and complied with NIH Guidelines for the Care and Use of Laboratory Animals.

### 2.2 EEG electrode implantation

Rats were anesthetized with 20% urethane (1 g/kg, i.p.) and positioned on a stereotaxic apparatus (RWD, China). A midline cranial incision was made to expose the anterior fontanel. Intracranial electrodes were implanted vertically at the following coordinates (relative to bregma): left occipital lobe (AP −4.80 mm, ML −3.0 mm), right occipital lobe (AP −4.80 mm, ML + 3.0 mm), and left parietal frontal lobe (AP + 2.0 mm, ML −2.0 mm). Electrodes were fixed with dental cement, and penicillin (i.p.) was administered to prevent infection. Rats were allowed 7 days of recovery before EEG recording and seizure induction.

### 2.3 EEG recording

EEG signals were recorded using a Solar 1848 system (sampling rate: 500 Hz; bandpass filter: 0.01–70 Hz). Recordings were synchronized with video monitoring to correlate electrophysiological changes with behavioral seizures. Epileptiform discharges (sharp waves, spike waves, polyspike waves) were quantified using Clampfit 10.7 software (Molecular Devices, United States).

### 2.4 Lithium-Pilocarpine seizure model and drug administration

Acute TLE was induced as previously described (Rashid et al., 2021; Zheng et al., 2022). Rats received lithium chloride (3 mmol/kg, i.p.) 18–20 h prior to pilocarpine injection. Scopolamine methyl nitrate (1 mg/kg, i.p.) was administered 30 min before pilocarpine to block peripheral cholinergic effects. Pilocarpine (40 mg/kg, i.p.; Med Chem Express, Cat. HY-B0726) was then injected to induce seizures. Seizure activity was monitored for 2 h, and Racine stage IV/V seizures were defined as generalized seizures (Racine, 1972). Diazepam (10 mg/kg, i.p.) was administered at the end of the 2 h period to terminate seizures and prevent fatal status epilepticus. Zingerone, dissolved in saline, was administered via intraperitoneal (i.p.) injection into the lower quadrant of the abdomen.

A total of 50 rats were randomly divided into five groups:

(a) Control group (Con, *n* = 8): Received saline instead of lithium chloride, pilocarpine, and zingerone. (b) Pilocarpine group (Pilo, *n* = 12): Received lithium-pilocarpine and saline (i.p.) at the onset of the first generalized seizure. (c) Zingerone low-dose group (ZO-L, *n* = 10): Received lithium-pilocarpine and zingerone (75 mg/kg, i.p.; Shanghai Macklin Biochemical Co., Ltd., V820486-5g) at the onset of the first generalized seizure. (d) Zingerone medium-dose group (ZO-M, *n* = 10): Received lithium-pilocarpine and zingerone (150 mg/kg, i.p.). (e) Zingerone high-dose group (ZO-H, *n* = 10): Received lithium-pilocarpine and zingerone (300 mg/kg, i.p.).

### 2.5 Brain tissue preparation

Seven days after seizure induction, rats were anesthetized with 20% urethane (1 g/kg, i.p.) and transcardially perfused with normal saline followed by 4% paraformaldehyde (PFA) in 0.1 M PBS (pH 7.2–7.4). Brains were extracted, post-fixed in 4% PFA overnight at 4°C, and dehydrated in 20–30% sucrose solution (in PBS) for 3 days. Coronal hippocampal slices (20 μm) were prepared using a cryostat (Leica, Germany) for IHC, HE staining, and immunofluorescence. The hippocampal slices (20 μm) used for all histological analyses (IHC, HE, Immunofluorescence) were collected from the dorsal hippocampus, specifically between −2.5 mm and −4.5 mm relative to bregma. In each animal, every 10th slice along the septotemporal axis of the hippocampus was included in the analysis. At least three sections were imaged for each animal. We have revised the text to state this clearly.

### 2.6 Immunohistochemistry

Slices were washed with PBS (0.01M, PH 7.2–7.4). Endogenous peroxidase blocking agent incubated at room temperature for 10 min, followed by PBS wash. Then incubated with 5% goat serum at room temperature for 30 min. We used the following primary antibodies as GFAP (1:1,000. CST, United States, Cat# 34001S) and IBa1 (1:1,000, CST, United States, Cat# 17198S), overnight incubation at 4°C following the protocol by Rashid et al. (2021) and Zheng et al. (2022). The next day, after washing three times with PBS, the sections were incubated with horseradish peroxidase (HRP)-conjugated secondary antibody at room temperature for 1 h. Immunoreactivity was visualized using 3,3′-diaminobenzidine (DAB, Max VisionIII Mouse/Rabbit). After immunoreactivity, slices were dipped in distilled water, dehydrated, mounted with neutral gum, and observed under an Olympus BX50 microscope. IBA1^+^ and GFAP^+^ cells in the hippocampal CA1 region were quantified using ImageJ software (NIH, United States).

### 2.7 Immunofluorescence staining

Slices were blocked with 5% goat serum for 30 min, incubated with anti-NeuN primary antibody (1:800, Abcam, Cat# ab104224) overnight at 4°C, and then with Alexa Fluor^®^488-conjugated secondary antibody (1:1,000, Abcam, Cat# ab150113) for 2 h at room temperature in the dark. Nuclei were stained with DAPI (1:1,000, Biosharp, P24000042957) for 5 min. Slices were mounted with 50% glycerol and imaged using a Nikon confocal laser scanning microscope. NeuN^+^ neuron density in the hippocampal CA1 region was quantified using Image J.

### 2.8 Hematoxylin and eosin staining

Slices were washed with PBS three times (5 min each), stained with hematoxylin for 3 min, rinsed with tap water until blue, differentiated with 1% hydrochloric acid ethanol for 2–3 s, and rinsed again with tap water for 5 min. Slices were then stained with eosin for 3 min, dehydrated in gradient ethanol (85, 95, 100%), cleared with xylene, and mounted with neutral gum. Neuronal morphology in the hippocampal CA1 region was observed under a Nikon optical microscope. Neurons exhibiting cytoplasmic shrinkage and hyperchromatic nuclei were identified as damaged cells. Quantification were expressed as the average number of pyknotic cells, three non-overlapping high-power field (HPF, 65 μm × 65 μm) were randomly selected per section.

### 2.9 Whole-cell patch-clamp recordings

Four-week-old male SD rats were anesthetized with isoflurane and decapitated. Brains were rapidly removed and immersed in ice-cold artificial cerebrospinal fluid (ACSF) containing (in mM): NaCl 124, KCl 2.5, NaH_2_PO_4_ 1.2, NaHCO_3_ 24, CaCl_2_ 2, MgCl_2_ 2, glucose 12.5, HEPES 5 (pH 7.35–7.45, osmolality 310–330 mOsm/L). Coronal brain slices (300 μm) containing the hippocampus were prepared using a vibrating microtome (Leica VT1000S) and incubated in oxygenated ACSF at 33°C for 30 min, then maintained at room temperature until recording.

Whole-cell patch-clamp recordings were performed on hippocampal CA1 pyramidal neurons using infrared differential interference contrast (IR-DIC) optics (Nikon FN1). The patch pipette solution contained (in mM): K-gluconate 140, HEPES 10, phosphocreatine 5, EGTA 0.5, Mg-ATP 4, Tris-GTP 1, NaCl 4 (pH 7.4, osmolality 290 mOsm/L). Action potentials were recorded using a Multiclamp 700B amplifier (Molecular Devices, United States) and sampled at 10 kHz with a Digidata 1550 interface. Data were analyzed using Clampfit 10.7 software (Molecular Devices).

### 2.10 Action potential parameter measurements

Inter-spike interval (ISI): The time interval between consecutive action potential peaks. The peak amplitude of action potentials (AP): Measured from the resting membrane potential baseline to the peak of the action potential. Threshold: Defined as the membrane potential level at which the rising phase slope exceeds dV/dt > 20 V/s. Afterhyperpolarization (AHP): The maximum hyperpolarizing deflection of the membrane potential below the resting baseline following an action potential (Figure 6C). Number of spikes: The total count of full action potentials recorded within a given time window.

### 2.11 Statistical analysis

Data were analyzed using OriginPro 2019 (OriginLab Corporation, United States) and GraphPad Prism 9 (GraphPad Software, Inc., United States). Comparisons between two groups were executed using either paired or unpaired Student’s *t*-tests. Multiple group comparisons, after passed Shapiro-Wilk test, were analyzed by the non-parametric Kruskal-Wallis H test or one-way ANOVA with Dunn’s *post-hoc* test, or two-way ANOVA with Sidak’s multiple comparisons test. The data was presented as the mean ± standard error of the mean (SEM), *p < 0.05 was considered statistically significant.

## 3 Results

### 3.1 Zingerone attenuates acute generalized seizures in TLE rats

**FIGURE 1:**
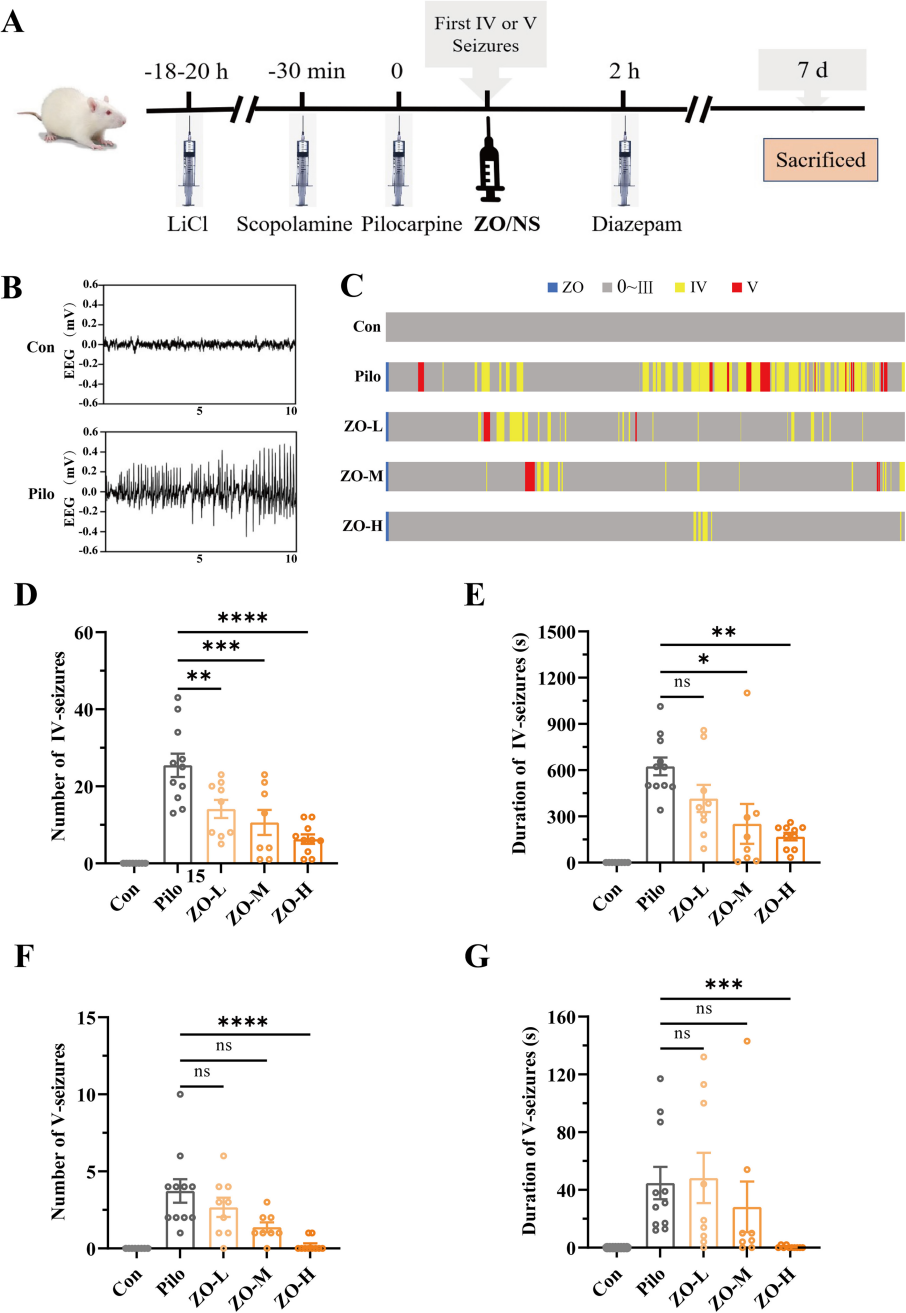
Zingerone attenuates acute generalized seizures in lithium-pilocarpine-induced TLE rats. **(A)** Schematic of the experimental timeline. **(B)** Representative EEG traces: The control group shows stable background activity; the pilocarpine group exhibits high-amplitude sharp waves, spike waves, and polyspike waves. **(C)** Representative plot of seizure activity: The graph illustrates seizure activity within 2 h from the first generalized seizure. Each cluster of graph corresponds to an experimental group (Con, Pilo, ZO-L, ZO-M, ZO-H), within a cluster representing the cumulative duration or times of seizures at stages 0 through V. The blue bar represents the timing of zingerone administration. The gray bar represents the seizures below Racine III (or no seizure, as in the normal group). While yellow and red bars indicate the stage 4 and 5 seizures, respectively. **(D)** Quantification of Racine IV seizure times during 2 h: ZO-M and ZO-H significantly reduced seizure times compared to the pilocarpine group. (E) Quantification of Racine IV seizure total duration during 2 h: ZO-M and ZO-H significantly shortened seizure duration. **(F)** Quantification of Racine V seizure: ZO-H significantly reduced seizure times. **(G)** Quantification of Racine V seizure total duration: Only ZO-H significantly shortened seizure duration. Data are presented as mea ± SEM. *p < 0.05, **p < 0.01, ***p < 0.001, ****p < 0.0001 vs. pilocarpine group (the non-parametric Kruskal-Wallis H test with Dunn’s *post-hoc* test).

Lithium-pilocarpine injection successfully induced acute TLE in rats. The pilocarpine group exhibited frequent Racine IV/V generalized seizures over the 2 h monitoring period, accompanied by EEG epileptiform discharges (sharp waves, spike waves, and poly spike waves) (Figure 1B). As illustrated in Figure 1A, the experimental timeline included lithium chloride pretreatment, scopolamine administration, pilocarpine-induced seizure induction, zingerone/saline injection at the first generalized seizure, and 2 h behavioral/EEG recording followed by diazepam termination.

Behavioral analysis confirmed the anticonvulsant effect of zingerone. For Racine IV seizures, the pilocarpine group experienced 25.45 ± 3.02 times and lasted a total seizures duration of 624.27 ± 57.93 s during 2 h (*n* = 11). ZO-M (150 mg/kg) significantly reduced these to 10.63 ± 2.78 times (*p* = 0.0003) and 251.38 ± 109.94 s (*p* = 0.0196, *n* = 8), while ZO-H (300 mg/kg) further decreased them to 6.3 ± 1.19 times (*p* < 0.0001) and 168.5 ± 23.09 s (*p* = 0.0049, *n* = 10) (Figures 1C–E). The ZO-L group (75 mg/kg) reduced the number to 4.11 ± 2.12 times (*p* < 0.0043, *n* = 9) but not alter duration (416.11 ± 79.44 s, *p* = 0.78).

For Racine V seizures, the pilocarpine group had a number of 3.73 ± 0.76 times lasting 44.73 ± 12.32 s during 2 h. ZO-H significantly decreased both measures (0.2 ± 0.14 times, *p* < 0.0001; 0.4 ± 0.28 s, *p* < 0.0002) (Figures 1F,G). ZO-M showed a non-significant reduction (1.38 ± 0.31 times; 28.25 ± 16.53 s), and ZO-L had no effect (*p* > 0.05). As shown in Figure 1C, the distribution of seizure severity was assessed over a 2 h period using a grouped bar chart according to the Racine scale.

### 3.2 Zingerone inhibits hippocampal neuroinflammation

To investigate whether zingerone exerts neuroprotective effects, rat brain tissues were collected 7 days after the establishment of the lithium chloride-pilocarpine-induced acute seizure. Immunohistochemical staining was performed to examine morphological changes in microglia, astrocytes, and neurons in the hippocampal CA1 region-a key area involved in TLE pathogenesis.

**FIGURE 2:**
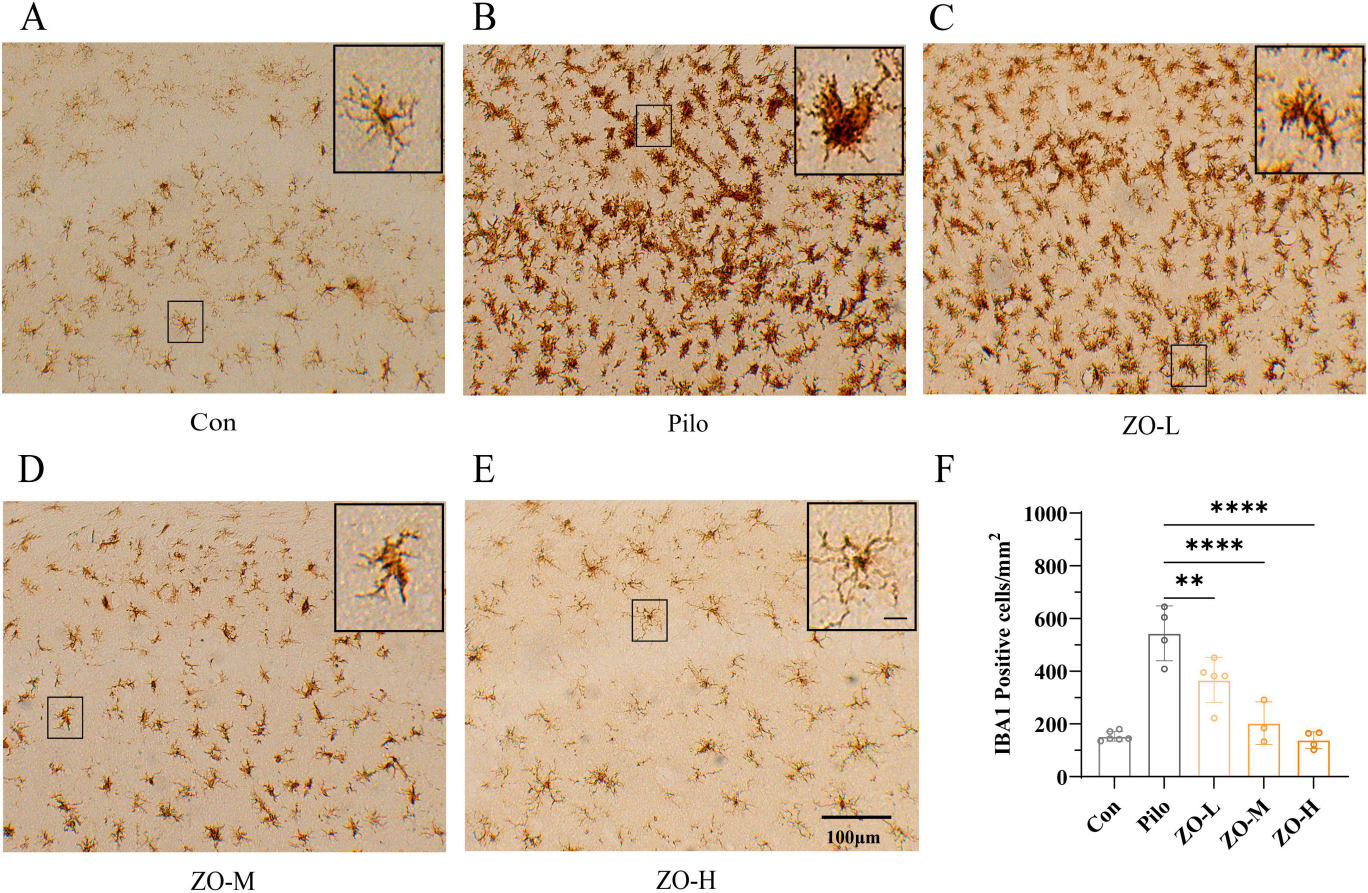
Zingerone inhibits microglial activation in the hippocampal CA1 region of TLE rats. **(A)** Representative IHC staining of IBA1 in the Con group: Resting microglia with small cell bodies and long, thin processes (scale bar: 100 μm). **(B)** Representative IHC staining of IBA1 in the Pilo group: Activated microglia with hypertrophied cell bodies and shortened, thickened processes (*n* = 4, scale bar: 100 μm). **(C–E)** Representative IHC staining of IBA1 in ZO-L, ZO-M, and ZO-H groups: Dose-dependent reduction in microglial activation (scale bar: 100 μm). **(F)** Quantification of IBA1^+^ cell density: ZO-M and ZO-H significantly reduced IBA1^+^ cell density compared to the Pilo group. Data are presented as mean ± SEM. **p < 0.01, ****p < 0.0001 [one-way ANOVA with Dunn’s *post-hoc* test, *F*(4, 17) = 27.23].

For microglial activation (marked by IBA1), the control group showed resting microglia with small cell bodies and long, thin processes (Figure 2A). In contrast, the pilocarpine group exhibited robust microglial activation, characterized by hypertrophied cell bodies, shortened and thickened processes, and a significant increase in IBA1^+^ cell density (544 ± 52.19 cells/mm^2^, *n* = 4, vs. Con: 153 ± 7.36 cells/mm^2^, *n* = 6, *p* < 0.0001) (Figures 2B,F). Zingerone treatment dose-dependently reversed these changes: ZO-M reduced IBA1^+^ cell density to 203 ± 46.48 cells/mm^2^ (*n* = 3, *p* < 0.0001 vs. Pilo) with partial restoration of microglial morphology, while ZO-H further decreased density to 139 ± 15.93 cells/mm^2^ (*n* = 4, *p* < 0.0001 vs. Pilo) with morphology nearly identical to the Con group (Figures 2D–F). The ZO-L group showed minimal improvement (IBA1^+^ density: 367 ± 38.28 cells/mm^2^, *n* = 5, *p* < 0.01 vs. Pilo) (Figures 2C,F).

**FIGURE 3:**
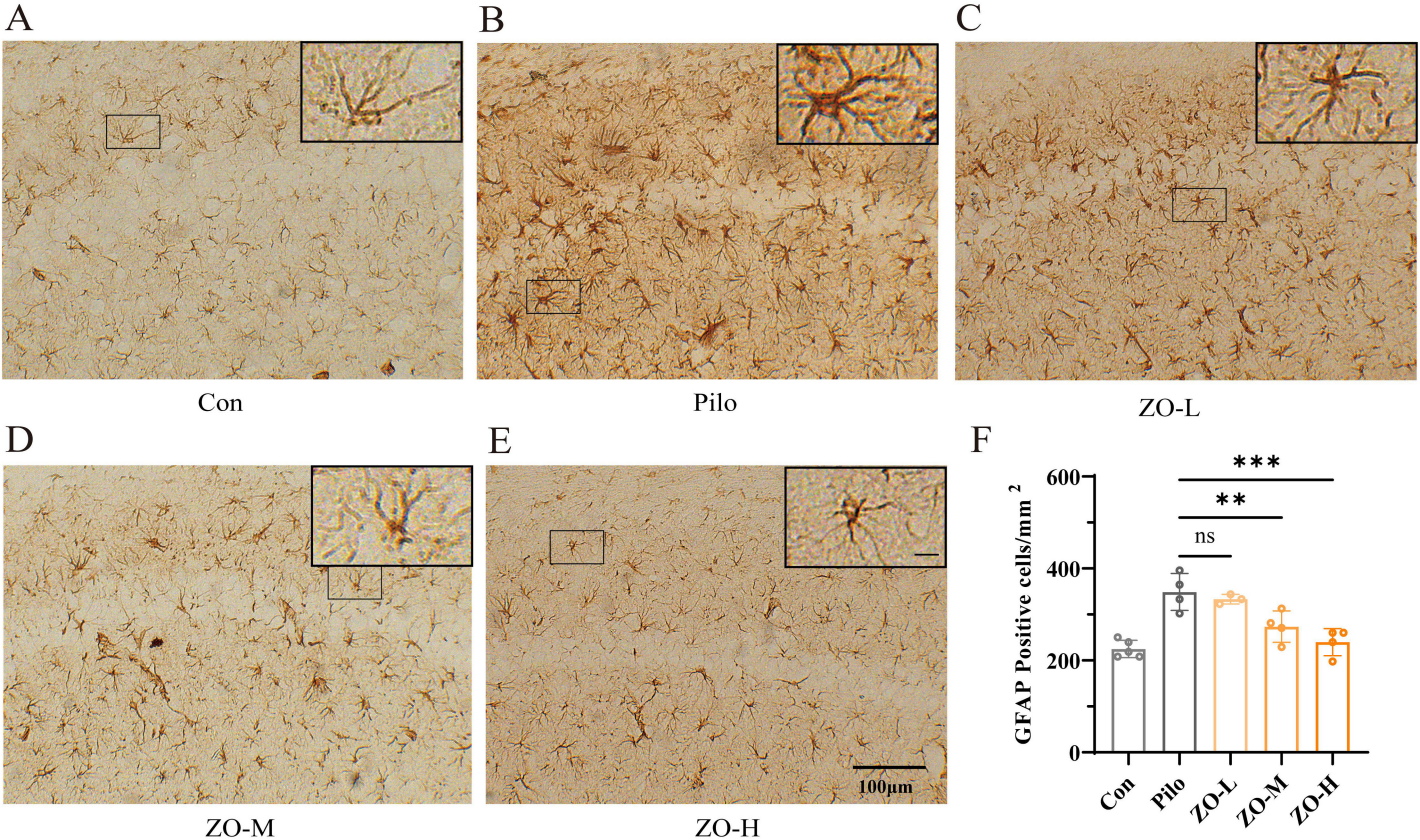
Zingerone inhibits astrocytic activation in the hippocampal CA1 region of TLE rats. **(A)** Representative IHC staining of GFAP in the Con group: Quiescent astrocytes with thin, ramified processes (scale bar: 100 μm). **(B)** Representative IHC staining of GFAP in the Pilo group: Activated astrocytes with hypertrophied cell bodies and thickened processes (scale bar: 100 μm). **(C–E)** Representative IHC staining of GFAP in ZO-L, ZO-M, and ZO-H groups: Dose-dependent reduction in astrocytic activation (scale bar: 100 μm). **(F)** Quantification of GFAP^+^ cell density: ZO-M and ZO-H significantly reduced GFAP^+^ cell density compared to the Pilo group. Data are presented as mean ± SEM [*F*(4, 15) = 14.48, *p* < 0.0001]. **p < 0.01, ***p < 0.001 vs. Pilo group (one-way ANOVA with Dunn’s *post-hoc* test).

For astrocytic activation (marked by GFAP), the Con group had quiescent astrocytes with thin, ramified processes (Figure 3A). The pilocarpine group displayed prominent astrocytic activation, with hypertrophied cell bodies, thickened processes, and increased GFAP^+^ cell density (*n* = 4, 349 ± 20.17 cells/mm^2^ vs. Con: 225 ± 8.46 cells/mm^2^, *n* = 5, F(4,15) = 14.58, *p* < 0.0001) (Figures 3B,F). Zingerone treatment inhibited astrocytic activation in a dose-dependent manner: ZO-M reduced GFAP^+^ cell density to 273 ± 17.21 cells/mm^2^ (*n* = 4, *p* = 0.0078 vs. Pilo) and improved astrocyte morphology, while ZO-H decreased density to 240 ± 14.73 cells/mm^2^ (*n* = 4, *p* = 0.0003 vs. Pilo) with near-normal morphology (Figures 3D–F). The ZO-L group showed no significant reduction in GFAP^+^ density (333 ± 6.01 cells/mm^2^, *n* = 3, *p* > *0.05* vs. Pilo) (Figures 3C,F).

### 3.3 Zingerone exerts neuroprotective effects in the hippocampus

HE staining and NeuN immunofluorescence were used to evaluate neuronal damage and survival in the hippocampal CA1 region. The experimental results showed that the effect of zingerone was dose-dependent.

**FIGURE 4:**
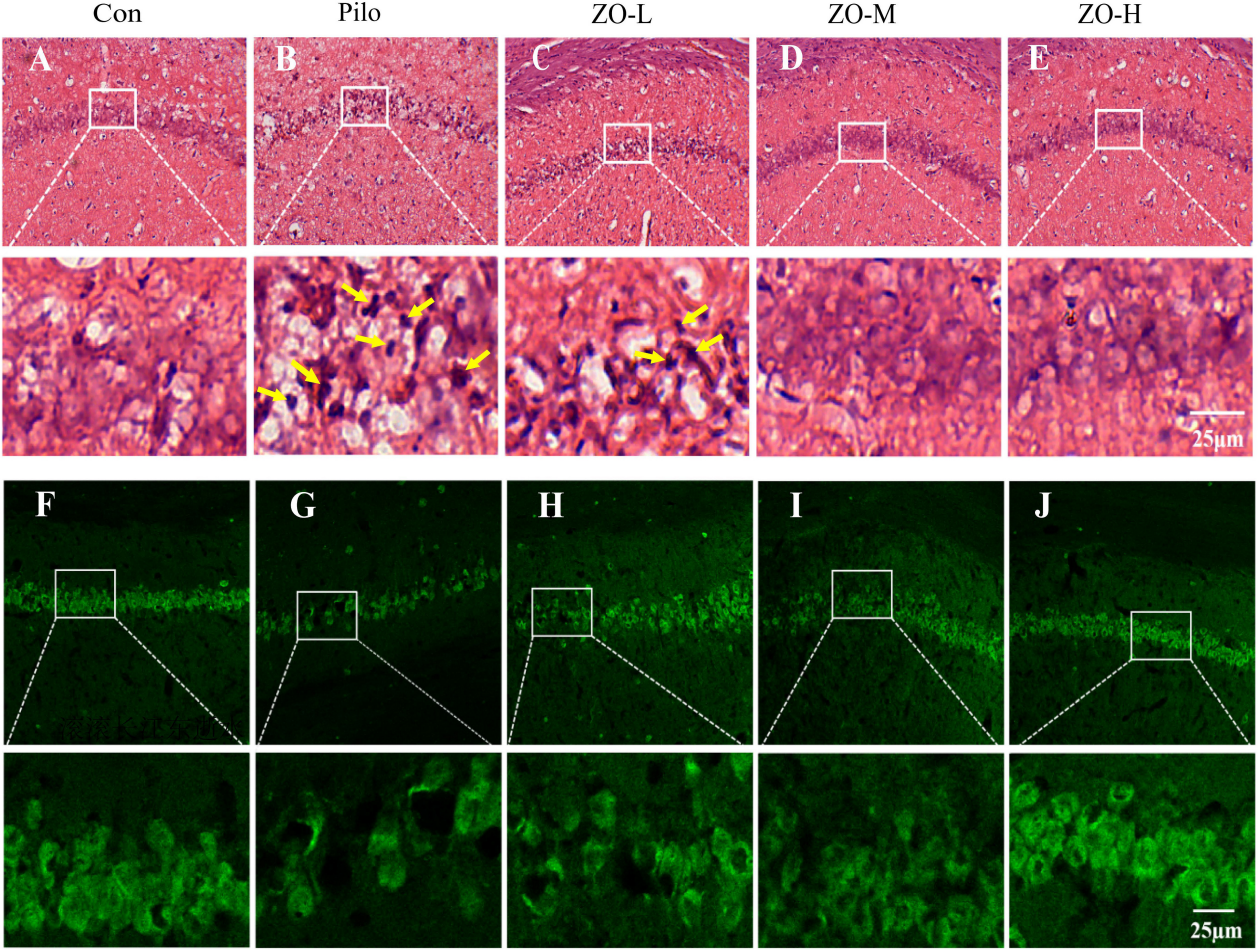
Zingerone exerts neuroprotective effects in the hippocampal CA1 region of TLE rats. **(A–E)** Representative HE staining images: **(A)** Con group: Intact neuronal morphology with round cell bodies and distinct nucleoli. **(B)** Pilo group: Shrunken cell bodies, pyknotic nuclei, and loss of nucleoli, as indicated by the yellow arrow. **(C)** ZO-L group: Minimal improvement in neuronal morphology. **(D)** ZO-M group: Moderate preservation of neuronal morphology. **(E)** ZO-H group: Near-normal neuronal morphology. **(F–J)** Representative NeuN immunofluorescence images (green: NeuN). **(F)** Con group: High density and orderly arrangement of NeuN^+^ neurons. **(G)** Pilo group: Reduced NeuN^+^ neuron density and disrupted architecture. **(H)** ZO-L group: exhibited a limited neuroprotective effect compared to the Pilo group. **(I)** ZO-M group: Increased NeuN^+^ neuron density. **(J)** ZO-H group: Near-normal density and distribution of NeuN^+^neurons, closely resembling the control group.

HE staining showed that the control group had intact neuronal morphology, with round, uniform cell bodies, distinct nucleoli, and tightly arranged layers (Figure 4A). The pilocarpine group exhibited severe neuronal damage, including shrunken cell bodies, condensed and deeply stained nuclei, loss of nucleoli, and disorganized neuronal layers (Figure 4B). Zingerone treatment dose-dependently preserved neuronal integrity: ZO-M showed moderate protection with relatively intact cell bodies and nucleoli (Figure 4D), while ZO-H exhibited near-normal neuronal morphology, with cell bodies and nucleoli similar to the Con group (Figure 4E). The ZO-L group showed minimal improvement in neuronal morphology (Figure 4C). Quantitative analysis of pyknotic neurons in HE-stained sections confirmed these observations. The pilocarpine group showed a significant increase in the number of pyknotic cells compared to the control group (Pilo: 20.60 ± 1.01 cells/HPF vs. Con: 3.11 ± 0.2 cells/HPF, *p* < 0.0001). Zingerone treatment dose-dependently reduced this parameter: ZO-L (16.95 ± 1.53 cells/HPF, *p* = 0.0497 vs.Pilo), ZO-M (8.61 ± 0.76 cells/HPF, *p* < 0.0001 vs. Pilo), and ZO-H (5.93 ± 0.49 cells/HPF, *p* < 0.0001 vs. Pilo). Data are presented as mean ± SEM [*F*(4, 24) = 54.35, *p* < 0.0001]. *p < 0.05, ****p < 0.0001 vs. Pilo group (one-way ANOVA with Dunn’s *post-hoc* test).

NeuN immunofluorescence (a marker for mature neurons) confirmed these findings. The control group had a high density of NeuN^+^ neurons with strong fluorescence intensity (Figure 4F). The pilocarpine group had a significant reduction in NeuN^+^ neuron density and weakened fluorescence (Figure 4G). ZO-M increased NeuN^+^ density vs. Pilo (Figure 4I), while ZO-H further restored density with fluorescence intensity comparable to the Con group (Figure 4J). The ZO-L group showed no significant increase in NeuN^+^ density (Figure 4H).

Collectively, these results demonstrate that zingerone provides substantial neuroprotection against seizure-induced hippocampal damage.

### 3.4 Zingerone modifies synaptic transmission within the hippocampal CA1

**FIGURE 5:**
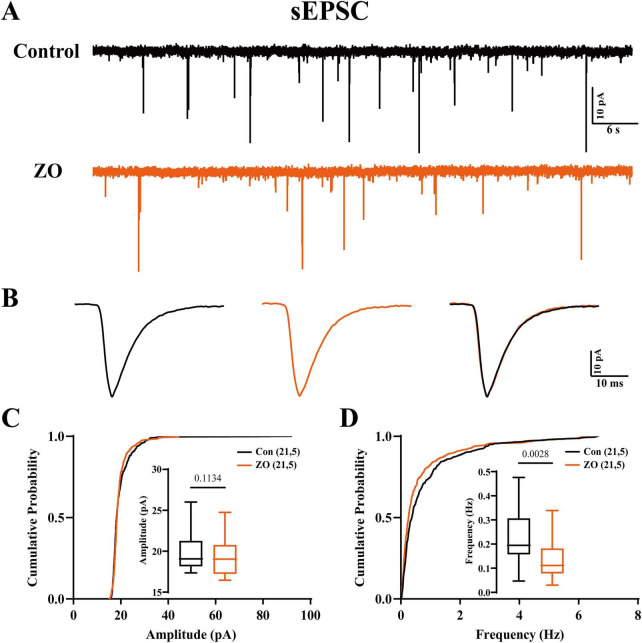
Zingerone modulates excitatory synaptic transmission in the CA1 pyramidal neurons. **(A)** Representative excitatory postsynaptic current (EPSC) traces recorded from hippocampal CA1 pyramidal neurons before and after application of zingerone. **(B)** The influence of zingerone on the amplitude of excitatory postsynaptic potentials (EPSPs) in CA1 pyramidal neurons is presented (orange curve). **(C)** The cumulative amplitude distribution and mean amplitude of EPSCs in ACC pyramidal neurons are also analyzed before and after the application of zingerone. **(D)** The cumulative frequency distribution and mean frequency of EPSCs in CA1 pyramidal neurons are presented, comparing measurements taken prior to and following zingerone (10 μM) administration. Control (black) and ZO (orange) groups are shown.

To elucidate the mechanisms by which zingerone modulates the activity of the CA1 pyramidal neurons, we employed whole-cell patch clamp techniques to assess the synaptic inputs to these neurons. Our findings indicate that zingerone (10 μM) administration resulted in an increased frequency of excitatory postsynaptic currents (EPSCs) in CA1 pyramidal neurons (Figures 5A,D, control: 0.22 ± 0.03 Hz, ZO: 0.14 ± 0.02 Hz, 21 cells from 5 mice, paired *t*-test, *p* = 0.0028), while the amplitude of EPSCs remained constant (Figures 5B,C, control: 19.78 ± 0.47 pA, ZO: 19.27 ± 0.50 pA, 21 cells from 5 mice, paired *t*-test, *p* = 0.1134). These results suggest that zingerone has the capacity to modulate the frequency of EPSCs in the hippocampal CA1 pyramidal neurons.

### 3.5 Zingerone reduces intrinsic excitability of hippocampal CA1 pyramidal neurons

Whole-cell patch-clamp recordings were performed to investigate the effects of zingerone on the electrophysiological properties of hippocampal CA1 pyramidal neurons. Zingerone (10 μM) was applied to brain slices via perfusion, and action potential parameters were analyzed.

**FIGURE 6:**
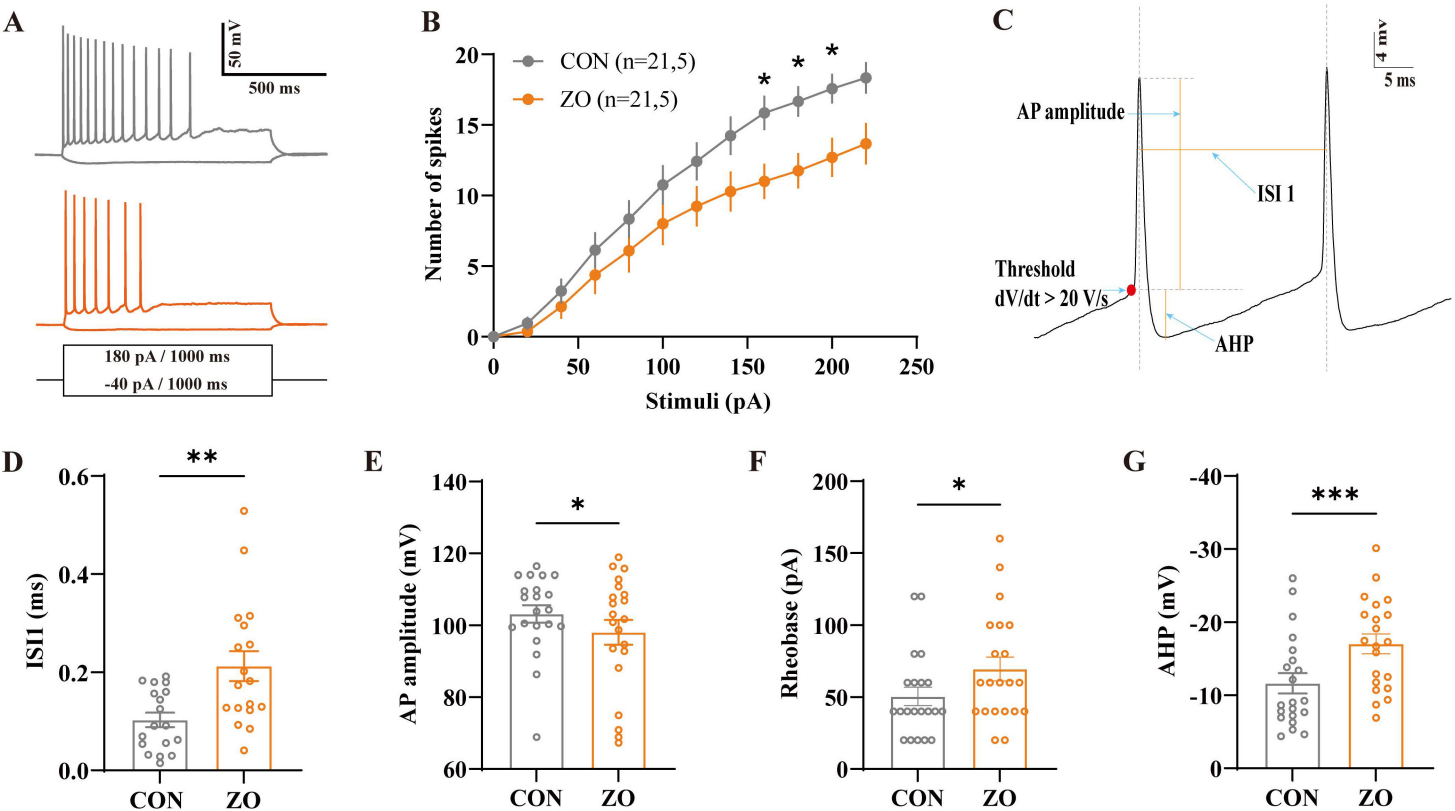
Zingerone reduces the intrinsic excitability of hippocampal CA1 pyramidal neurons. **(A)** Representative action potential traces in response to a depolarizing current pulse. The ZO group shows reduced firing frequency. The firing patterns are depicted without zingerone (black, top) and with zingerone (orange, middle) in response to the stimulus waveform (black, bottom). **(B)** The application of zingerone (10 μM) resulted in a rightward shift and a reduced gain in the input-output curve (repeated measures two-way ANOVA with Sidak’s multiple comparisons test between groups). **(C)** Schematic illustration depicting a typical neuronal action potential waveform and defining the key parameters measured. **(D)** The ZO group (orange trace) shows prolonged inter-spike interval (ISI) compared to the Con group(black trace) (unpaired *t*-test, *p* = 0.0026). **(E)** The effect of zingerone on the action potential peak is shown, demonstrating a reduction in the action potential peak (orange trace, paired Student’s *t*-test, *p* = 0.0416). **(F)** Zingerone increased the minimum current stimulation intensity required to elicit an action potential of pyramidal neurons (orange trace, paired Student’s *t*-test, *p* = 0.0423). **(G)** Afterhyperpolarization (AHP) amplitude: Zingerone (orange bar) significantly increased the AHP amplitude. *p < 0.05, **p < 0.01, ***p < 0.01 vs. Con group (paired Student’s *t*-test). Con, control; ZO, zingerone (10 μM); AHP, afterhyperpolarization.

Representative action potential traces showed that zingerone significantly reduced the firing frequency of CA1 neurons in response to depolarizing current pulses (Figures 6A,B). At a depolarizing stimulus of 160 pA, the firing frequency was 15.86 ± 1.21 Hz in the Control group, which decreased to 11 ± 1.26 Hz in the ZO group (Figure 6B, 21 cells from five mice, two-way ANOVA with Sidak’s multiple comparisons test between groups, *p* = 0.047). Further analysis revealed that zingerone prolonged the inter-spike interval (ISI) between the first and second action potentials: from 0.10 ± 0.01 ms (Con) to 0.21 ± 0.03 ms (ZO, *p* = 0.0026) (Figure 6D). Zingerone also slightly reduced the peak amplitude of action potentials (Con: 103.21 ± 2.44 mV; ZO: 98.10 ± 3.48 mV, *p* = 0.0416) (Figure 6E). Zingerone increased the minimum current stimulation intensity required to elicit an action potential of pyramidal neurons (Con: 50.48 ± 6.42; ZO: 69.52 ± 8.35, *p* = 0.0423) (Figure 6F). Additionally, Zingerone modulated other electrophysiological parameters: it increased the amplitude of the afterhyperpolarization (AHP) from −11.65 ± 1.38 mV (Con) to −17.06 ± 1.36 mV (ZO, *p* = 0.0001) (Figure 6G), indicating enhanced potassium channel function. These results indicate that zingerone reduces the intrinsic excitability of hippocampal CA1 neurons by prolonging the ISI, increasing AHP amplitude, and decreasing firing frequency.

## 4 Discussion

This study demonstrates that acute administration of zingerone exerts significant anticonvulsant and neuroprotective effects in a lithium-pilocarpine-induced temporal lobe epilepsy rat model. The therapeutic effects are primarily mediated through two key mechanisms: inhibition of hippocampal neuroinflammation and reduction of intrinsic neuronal excitability. These findings address an important gap in the existing literature and highlight that zingerone holds promise as a natural adjuvant therapy for drug-resistant temporal lobe epilepsy.

The acute anticonvulsant effect of zingerone during acute seizure episodes is particularly noteworthy, while previous research has predominantly focused on chronic administration or other ginger-derived compounds (Rashid et al., 2021; Kim et al., 2010, 2022b). Our study found that a single dose of zingerone (150–300 mg/kg) administered during generalized seizures significantly reduced the frequency and duration of Racine stage IV/V seizures without apparent adverse effects. This finding carries important clinical implications, as acute seizure management, especially in patients with drug-resistant epilepsy remains an unmet need, and existing treatments such as benzodiazepines are often associated with sedation and cognitive impairment (Lyman and Chetkovich, 2019). The dose-dependent effect of zingerone, with higher concentrations yielding better anticonvulsant outcomes, aligns with its known pharmacological properties: zingerone modulates neuronal excitability and inflammatory pathways in a concentration-dependent manner (Kudryashov et al., 2007; Molavinia et al., 2024; Eriten et al., 2024; Tuncer et al., 2024; Olasehinde and Olaokun, 2025; Xu et al., 2025; Oviosun et al., 2025). The robust central effects observed in our study, along with recent reports of zingerone’s neuroprotective actions in models of morphine dependence (Molavinia et al., 2024) and cognitive disorders (Olasehinde and Olaokun, 2025), strongly suggest that systemically administered zingerone can reach the brain at therapeutically relevant concentrations.

Neuroinflammation is a central driver in the progression of temporal lobe epilepsy, where activation of microglia and astrocytes promotes neuronal hyperexcitability and loss (Vezzani et al., 2016; Zhang et al., 2022). Immunohistochemical results show that zingerone dose-dependently suppressed the activation of IBA1^+^ microglia and GFAP^+^ astrocytes in the hippocampal CA1 region. The activated glial cells can release pro-inflammatory cytokines such as IL-1β and TNF-α, disrupting the excitatory inhibitory balance by enhancing glutamatergic transmission and inhibiting GABAergic signaling ( Ramos-Moreno et al., 2024; Rana and Musto, 2018; Borowicz-Reutt and Czuczwar, 2020).zingerone exerts multi-target anti-inflammatory effects in the brain. Specifically, zingerone has been shown to inhibit the NF-κB signaling pathway, downregulate pro-inflammatory cytokines (including IL-6 and TNF-α), and modulate oxidative stress (Hsiang et al., 2015; Rashid et al., 2021; Olasehinde and Olaokun, 2025; Mehrzadi et al., 2021). By reducing glial activation, zingerone may help to restore normal network activity. It is important to note, however, that reactive gliosis is a complex and context-dependent process. While our study focuses on the pro-inflammatory aspects of microglial and astrocytic activation following acute seizures, we acknowledge that glial cells can also exert beneficial effects in tissue repair and reorganization ( Vezzani et al., 2016). The observed increase in IBA1^+^ and GFAP^+^ cells in the pilocarpine group, characterized by hypertrophied morphologies, likely represents a shift toward a pro-inflammatory phenotype (often referred to as M1-like microglia and A1-like astrocytes) associated with neuronal injury. Future studies employing specific markers to differentiate between pro-inflammatory (e.g., CD68, iNOS for microglia; C3 for astrocytes) and anti-inflammatory (e.g., Arg1, CD206 for microglia; S100A10 for astrocytes) phenotypes would provide deeper insights into the nuanced modulatory effects of zingerone on glial function. This anti-inflammatory mechanism is further supported by zingerone’s neuroprotective effects: HE staining and NeuN immunofluorescence revealed that zingerone preserved neuronal morphology and reduced neuronal loss in the hippocampal CA1 region. Given that hippocampal sclerosis is a key pathological feature of temporal lobe epilepsy and contributes to drug resistance ( Patel et al., 2019), zingerone’s ability to protect hippocampal neurons may help slow disease progression.

The reduction of intrinsic neuronal excitability represents another key mechanism underlying zingerone’s anticonvulsant action. In TLE, CA1 pyramidal neurons exhibit hyperexcitability driven by ion channel dysfunction, altered synaptic plasticity, and neuronal network reorganization (Kudryashov et al., 2007; Yang et al., 2018). Our electrophysiological recordings demonstrate that zingerone decreases firing frequency, prolong ISIs, reduces AP amplitude, increases rheobase, and enhances AHP. Furthermore, zingerone was found to modify synaptic transmission to CA1 neurons. These effects are consistent with reported inhibition of voltage-gated sodium channels and potentiation of potassium currents (Kudryashov et al., 2007; Wang et al., 2025; Lai et al., 2022; Kim et al., 2022a). Inhibiting sodium channels reduces the influx of sodium ions, thereby limiting depolarization and action potential generation, while activating potassium channel enhances potassium efflux, and strengthens afterhyperpolarization, thereby reducing the likelihood of repetitive neuronal firing. Notably, zingerone did not alter AP threshold, indicating that it modulates firing patterns rather than the initiation of action potentials. This mechanism differs from that of traditional antiseizure medications such as carbamazepine, which primarily block sodium channels to raise the action potential threshold (Ryvlin et al., 2026). This distinct mode of action may partly explain zingerone’s efficacy in a drug-resistant temporal lobe epilepsy model, where ion channel alterations often render conventional drugs ineffective (Kwan et al., 2010).

Whether zingerone’s anti-inflammatory effect is a direct action on glial cells or an indirect consequence of its neuroprotective and anti-excitatory effects on neurons remains an open question. Our *in vivo* data cannot distinguish between these possibilities. It is plausible that both mechanisms contribute: zingerone may directly inhibit pro-inflammatory signaling pathways in glia, while simultaneously reducing neuronal hyperexcitability and injury, thereby diminishing the release of damage-associated molecular patterns (DAMPs) that trigger glial activation. Future studies employing *in vitro* co-culture systems, such as primary neuron-glia cultures, where zingerone can be applied in the presence or absence of neuronal activity blockers, will be essential to dissect the primary cellular targets of its anti-inflammatory action.

The novelty of this study lies in the simultaneous demonstration of zingerone’s dual modulation of neuroinflammation and neuronal excitability, two interconnected pathophysiological pathways in TLE. Most existing antiseizure medications target a single pathway, which may contribute to their limited efficacy in patients with drug-resistant epilepsy (Borowicz-Reutt and Czuczwar, 2020). Zingerone’s multi-target properties make it a promising candidate for addressing the complex pathological network of temporal lobe epilepsy. Moreover, as a low-toxicity, edible natural compound, zingerone is suitable for long-term adjuvant therapy (Li et al., 2019; Songvut et al., 2024). Studies by Songvut et al. (2024) have also provided pharmacokinetic evidence for the oral absorption and interconversion of ginger constituents, supporting the premise that orally administered ginger-derived compounds can yield systemic zingerone. Furthermore, recent work on nano-encapsulation of natural products (C Sekhar et al., 2024; Sekhar et al., 2025; Kunjumon et al., 2022) demonstrates how formulation strategies can enhance the bioavailability and neuroprotective efficacy of such compounds, which could be a promising approach for future development of zingerone-based therapies. In contrast to synthetic antiseizure medications, which are often associated with adverse effects such as hepatotoxicity and cognitive impairment (Ali et al., 2008; Ezzat et al., 2018), zingerone did not induce significant behavioral abnormalities in this study, suggesting a favorable safety profile.

## 5 Limitations of the study

Several important limitations of this study should be acknowledged, which also outline directions for future research:

### 5.1 Molecular targets

While our electrophysiological data demonstrate that zingerone reduces intrinsic neuronal excitability by modulating firing patterns, rheobase, and AHP, the precise molecular targets remain incompletely defined. Although our data suggest involvement of voltage-gated sodium and potassium channels, the specific channel subtypes (e.g., Nav1.1, Nav1.6; Kv7/KCNQ, BK, SK channels) await identification. Future investigations employing pharmacological blockade experiments in voltage-clamp mode, single-channel recordings, and molecular approaches (such as gene knockdown or knockout) are required to pinpoint the exact ion channel subunits targeted by zingerone.

### 5.2 Chronic efficacy and antiepileptogenesis

This study focused on acute anticonvulsant effects in an acute seizure model. The potential antiepileptogenic properties of zingerone and its effectiveness in chronic TLE models (e.g., spontaneous recurrent seizure models following status epilepticus) remain to be investigated. Long-term treatment studies are necessary to determine whether zingerone can modify disease progression, not just suppress acute seizures.

### 5.3 Toxicology and safety profile

While no overt adverse effects were observed in this acute study, a comprehensive toxicological evaluation of zingerone, particularly at the 300 mg/kg dose, is lacking. Future studies must assess its potential effects on motor function (e.g., rotarod test), cognition (e.g., Morris water maze), and major organ systems (e.g., liver and kidney histology and serum biochemistry) following both acute high-dose and chronic administration to define its therapeutic index.

### 5.4 Sex as a biological variable

This study used only male rats to minimize hormonal variability. However, given the well-documented sex differences in epilepsy (e.g., catamenial epilepsy) and potential sex-dependent drug responses, future studies must include both male and female animals to evaluate potential sex differences in zingerone’s efficacy and pharmacokinetics.

## 6 Conclusion

In summary, acute zingerone administration alleviates seizures and protects hippocampal neurons in a TLE rat model by suppressing neuroinflammation and reducing intrinsic excitability and synaptic transmission in CA1 pyramidal neurons. These results position zingerone as a promising natural compound for developing novel adjuvant therapies against drug-resistant TLE. Future research should explore its long-term efficacy in chronic epilepsy models, its potential antiepileptogenic properties, its precise molecular targets, oral bioavailability, comprehensive toxicological profile, and clinical translatability, with careful consideration of sex as a biological variable.

## Data Availability

The original contributions presented in this study are included in the article/supplementary material, further inquiries can be directed to the corresponding authors.
